# Different Phenotypes of Mature Biofilm in *Flavobacterium psychrophilum* Share a Potential for Virulence That Differs from Planktonic State

**DOI:** 10.3389/fcimb.2017.00076

**Published:** 2017-03-15

**Authors:** Héctor A. Levipan, Ruben Avendaño-Herrera

**Affiliations:** ^1^Laboratorio de Patología de Organismos Acuáticos y Biotecnología Acuícola, Facultad de Ciencias Biológicas, Universidad Andres BelloViña del Mar, Chile; ^2^Interdisciplinary Center for Aquaculture ResearchConcepción, Chile; ^3^Centro de Investigación Marina QuintayValparaíso, Chile

**Keywords:** *Flavobacterium psychrophilum*, bacterial cold water disease, rainbow trout fry syndrome, atlantic salmon, *Oncorhynchus mykiss*, virulence factors, biofilm formation, gene transcription

## Abstract

*Flavobacterium psychrophilum* is the etiological agent of bacterial coldwater disease and the rainbow trout fry syndrome in salmonid aquaculture worldwide. However, there have been few studies into the capacity of *F. psychrophilum* to form biofilms and how these cellular accretions differ from planktonic cells or how they affect potential virulence. We evaluated the biofilm formation by three Chilean isolates of *F. psychrophilum* (LM-02-Fp, LM-06-Fp, and LM-13-Fp) and two non-Chilean strains (JIP02/86 and NCMB1947^T^), and compared biofilm and planktonic states to obtain insights into expression differences of virulence- and biofilm-related genes (VBRGs). Our findings are based on scanning confocal laser microscopy (SCLM) and LIVE/DEAD staining, enzymatic reactions, reverse transcription-quantitative PCR (RT-qPCR) of genes encoding putative virulence factors, and transcriptomes (RNA-Seq). The LM-02-Fp and NCMB1947^T^ strains were the strongest and weakest biofilm producers, respectively. The strong-biofilm producer showed different physiological cell states distributed in different layers of mature biofilms, whereas the NCMB1947^T^ biofilms consisted of cells arranged in a monolayer. WGA-binding exopolysaccharides would be the main components of their corresponding extracellular matrices. Transcriptomes of *F. psychrophilum* NCMB1947^T^ and LM-02-Fp were clustered by state (biofilm vs. planktonic) rather than by strain, indicating important state-dependent differences in gene expression. Analysis of differentially expressed genes between states identified putative VBRGs involved in polysaccharide biosynthesis, lateral gene transfer, membrane transport (e.g., for drugs and Fe^3+^), sensory mechanisms, and adhesion, and indicated that about 60–100% of VBRGs involved in these processes was significantly upregulated in the biofilm state. Conversely, upregulated motility-related genes in the biofilm state were not identified, whereas a lower fraction of proteolysis-related genes (33%) was upregulated in biofilms. In summary, *F. psychrophilum* strains that produce different biofilm phenotypes show global transcriptional activity in the mature biofilm state that differs significantly from their planktonic counterparts. Also, different biofilm phenotypes share a genetic potential for virulence that is transcriptionally enhanced with respect to free-living cells. Our results suggest that the *F. psychrophilum* biofilm lifestyle acts as a reservoir for a given set of putative virulence factors, and recommend a deeper understanding of which could help prevent recurring infections in salmonid farms.

## Introduction

Bacteria belonging to *Flavobacterium* spp. have life strategies that allow them to thrive in a variety of environments. At least four species are known to be pathogenic to fish (Bernardet and Bowman, 2006), including *Flavobacterium psychrophilum*, a Gram-negative and filamentous psychrophilic bacterium responsible for bacterial coldwater disease (BCWD) and rainbow trout fry syndrome (RTFS) in freshwater salmonid aquaculture (e.g., Lorenzen et al., 1997), but also affects non-salmonid freshwater fish (Elsayed et al., 2006; Verma and Prasad, 2014). BCWD produces lesions on the skin along with fin and spinal deformities in survivor fish, while RTFS is characterized by producing lethargy, disorientation, lack appetite in adults, and occasional epidermal lesions (Starliper, 2011). Since the first description of the disease in farmed rainbow trout (Davis, 1946), *F. psychrophilum* has become one of the most detrimental pathogens for global salmonid fish farming (Nematollahi et al., 2003a), making the infected fish more vulnerable to viral diseases (Evensen and Lorenzen, 1997; Avendaño-Herrera et al., 2009,^1^).

Completely sequenced chromosomes of *F. psychrophilum* (10 at the time of this publication^2^) have revealed several genes encoding proteins with a potential role in pathogenicity (Duchaud et al., 2007). Castillo et al. (2016) recently reported that *F. psychrophilum* harbors at least 3373 genes and a core genome minimum of 1743 genes, with putative virulence factors uniformly distributed among 11 isolates from different sources and locations (Castillo et al., 2016). Remarkably, all of these isolates show similar capacities to form biofilms, with implications for their virulence and pathogenicity. Indeed, biofilms are physiologically variable cell populations adhering to an interface (usually solid-liquid) through an extracellular polymeric substance that provides protection (e.g., against therapeutic drugs and host defense mechanisms), facilitates cell-to-cell communication (e.g., horizontal gene transfer), and acts as a seed-bank for dissemination of pathogens (Flemming et al., 2016).

*Flavobacterium psychrophilum* strains are known to attach themselves to and can form biofilms on living surfaces of fish bodies, spleen, gills, and eggs (Vatsos et al., 2001; Kondo et al., 2002; Nematollahi et al., 2003b; Strepparava et al., 2012), as well as on non-living surfaces such as polystyrene, stainless steel, and glass (Högfors-Rönnholm et al., 2014; Ríos-Castillo, 2014; Leyton et al., 2015), with possible involvement of pilli-like structures (Morgan et al., 2009)^1^, adhesins (Duchaud et al., 2007), and the O-polysaccharide (Papadopoulou et al., 2015). In addition, mature biofilms can develop resistance to oxytetracycline and flumequine after a brief exposure to sub-inhibitory concentrations of these drugs (Sundell and Wiklund, 2011). A study based on *F. psychrophilum* mutants with defects in motility (Álvarez et al., 2006) reported that these showed improved ability to form biofilms, although also with lower levels of extracellular protease activity, virulence, and cytotoxicity. Moreover, the adherence capacity of some virulent *F. psychrophilum* strains could be an interface-specific skill rather than virulence dependent (Møller et al., 2003), and hence unrelated to virulence (Vatsos et al., 2001). Nevertheless, other studies suggest a positive association between virulence and biofilm formation (Nematollahi et al., 2003b; Högfors-Rönnholm et al., 2014; Castillo et al., 2015). These apparent discrepancies underline the need for more biofilm studies.

Therefore, more insights are needed into the ecology and pathogenesis of *F. psychrophilum* to understand how this microorganism triggers infection in fish. The aim of this study was to characterize *F. psychrophilum* during biofilm development and determine the potential virulence of this phenotype (evaluated here through differential expression of putative virulence genes). Our results are based on a comparison of *F. psychrophilum* strains grown in biofilm and planktonic states to gain insight into expression differences of virulence- and biofilm-related genes (VBRGs). The results indicate that *F. psychrophilum* strains that produce different types of biofilm trigger a global transcriptional response in the biofilm mode that differs substantially from that of the planktonic state, but not among different strains. Moreover, different biofilm phenotypes share a potential for virulence (here represented by a given set of upregulated genes) that does not necessarily diminish with respect to free-living cells, as has been speculated.

## Materials and methods

### Experimental design and methodological considerations

This study used three Chilean isolates of *F. psychrophilum* from our private collection (LM-02-Fp, LM-06-Fp, and LM-13-Fp) and two non-Chilean strains (JIP02/86 and NCMB1947^T^). The Chilean strains have been described elsewhere (Solís et al., 2015) and were isolated from the kidneys of clinically infected rainbow trout (*Oncorhynchus mykiss*). The strain JIP02/86 was isolated in France (Bernardet and Grimont, 1989), while the NCMB1947^T^ strain was purchased from the American Type Culture Collection. The studied strains were confirmed as *F. psychrophilum* by 16S rDNA-based PCR analysis (see details below).

All strains were evaluated with the crystal violet (CV)-microtiter plate method to select strong and weak biofilm-formers. The biofilm characterization implied a trade-off between (1) samples collected at early stages of biofilm formation with more viable cells but that may not have been representative of those found in aquaculture settings, and (2) samples collected from mature biofilms composed of physiologically variable cells (i.e., active, inactive, and even dead), as would be expectable in aquaculture settings. We choose the second option so a specific point in time of maximum biofilm formation (i.e., 4-day-old biofilms) was characterized and compared against planktonic cells. The characterization was done by scanning confocal laser microscopy (SCLM) coupled with LIVE/DEAD staining, enzymatic profiling using a miniaturized reaction system, reverse transcription-quantitative PCR (RT-qPCR) of seven specific genes, and transcriptome sequencing (RNA-Seq) to investigate the global shift patterns in gene expression with a focus on VBRGs. In addition, detection of *N*-acyl homoserine lactones (AHLs) in cell-free culture supernatants was performed by gas chromatography-mass spectrometry. Since we could not obtain RNA samples with a RNA integrity number (RIN) acceptable for RNA-Seq from planktonic cells on 4-day-old biofilms (i.e., RIN score ≥6.3 in the current study), RNA-Seq data derived from these biofilms were compared with those generated from cultures in the exponential growth phase (i.e., 2-day-old cultures). RIN scores (which range between 1 and 10 for totally degraded and intact RNA, respectively) higher than 6.0 are generally considered acceptable for gene expression analysis (Takahashi et al., 2010; Liu et al., 2015). A detailed description of the experimental procedures is provided below.

### Storage of strains, culture conditions, and AHLs detection

For long-term storage of bacterial strains, log-phase cultures grown in tryptone yeast extract salts medium (TYES; Holt et al., 1993) were mixed with sterile glycerol (15%, final concentration) and 1 mL aliquots were frozen at −80°C in cryovials. All strains were routinely cultured with agitation (150 rpm) at 17 ± 1°C in half-strength TYES, and with up to two subcultures removed from the original stock at −80°C.

All strains of *F. psychrophilum* and a positive control strain for C6- to C12-AHLs production (*Yersinia ruckeri* CECT 955; Kastbjerg et al., 2007) were cultured under the aforementioned routine conditions and grown until the late exponential phase. A 1-mL aliquot was withdrawn from each culture and separately inoculated in half-strength sterile TYES broth (50 mL) that was then incubated for an additional 48 h. A standard was prepared containing *N*-hexanoyl- and *N*-decanoyl-DL-homoserine-lactones in 50 mL of half-strength sterile TYES (0.5 ng μL^−1^ of each AHL, final concentration; Sigma-Aldrich). Cell cultures and standard (50 mL) were filtered through 0.22 μm (MS® MCE Syringe Filters; Membrane solutions LLC, USA), acidified with HCl 1 N (to pH = 2.0), and kept stirring (200 rpm) overnight at room temperature. AHLs (in control and standard samples) and presumptive AHLs (in *F. psychrophilum* samples) were extracted with CH_2_Cl_2_ (3 × 100 mL). The organic phase was dried with anhydrous Na_2_SO_4_, filtered, and the solvent was evaporated using a rotary evaporator (Heidolph, Germany). The residues were dissolved in 0.5 mL of HPLC-grade acetonitrile and stored at −20°C until analysis by gas chromatography-mass spectrometry (Shimadzu, GC-17A, GCMS-QP5050A; Shimadzu Instruments, Columbia, MD). The GC was equipped with a fused silica RTX-5 capillary column (30 m × 0.25 mm id, 0.25 mm film, Restek, Bellefonte, PA, USA). Helium was used as the carrier gas and the GC was programmed from 60°C for 2 min to increase to 270°C at 10°C/min. The injector temperature was set at 200°C. Mass spectra were acquired at 70 eV.

### Biofilm development

Strong and weak biofilm-forming strains were identified with the CV-microtiter plate method (Niu and Gilbert, 2004), and one representative strain of each category was selected for further analysis. Briefly, 150 μL aliquots of culture were inoculated in quintuplicate for each strain into a 96-well microtiter polystyrene plate (flat-bottom Corning Costar® plates, NY, USA) at an average density of 2.15 ± 0.83 × 10^7^ CFU mL^−1^ (± SD). As well, 150 μL aliquots of sterile TYES broth (half-strength) were added in quintuplicate as negative controls along with the assayed strains. Four inoculated microplates were incubated with agitation (40 rpm) at 17 ± 1°C until they were processed individually at 24-h intervals to evaluate biofilm development over a 4-day period. To do so, inoculated and control wells were emptied, washed (3X) with 200 μL of sterile milli-Q water, and stained with 180 μL of CV solution (1% wt/vol, Winkler chemistry) at room temperature (~25°C) for 15 min. The CV solution was then discarded and the wells were washed (4X) with 200 μL of sterile milli-Q water by repeated pipetting. The microplate was turned upside down to dry at room temperature for 15 min, and 180 μL of absolute ethanol was added per well for CV solubilization for 10–15 min. Biofilm formation was determined by reading absorbance of solubilized CV at 585 nm using a Tecan Microplate Reader (Infinite 200 PRO, Männedorf, Switzerland) and the specific biofilm formation (SBF) index proposed by Niu and Gilbert (2004):

$$\begin{array}{l} SBF\;=\;\left(B-NC\right)/G \end{array}$$

where *B* is the amount of ethanol-solubilized CV released from biofilm cells, *NC* is the amount of ethanol-solubilized CV that adhered to the polystyrene surfaces in negative controls, and *G* is the absorbance of the cell supernatant.

The strongest and weakest biofilm producers (LM-02-Fp and NCMB1947^T^ strains, respectively) were also analyzed by the CV-microplate technique with samplings every 48 h for a 12-days period. In addition, the two strains were analyzed by classical optical microscopy, for which purpose, both strains were grown in half-strength TYES medium, and then inoculated into 6-well microtiter plates (flat-bottom TPP® plates, Switzerland) by adding 1.8 mL of culture per well in order to achieve an average density per strain of 1.77 ± 0.46 × 10^7^ CFU cm^−2^ (± SD). Afterwards, a 5-cm^2^ sterile glass slide was placed in each microplate well and then incubated with agitation (40 rpm) at 17 ± 1°C until slide collection and processing. A negative control consisting of a well containing 1.8 mL of sterile medium and a sterile glass slide was included in every microplate. Inoculated slides were randomly collected every 24 h, while the negative control slides were collected at the end of the total incubation time. Once collected, all slides were dipped (3X) in sterile milli-Q water, stained by immersion in a CV solution (1% wt/vol) between 10 and 15 min, dipped again (3X) in sterile milli-Q water, and left to air-dry at room temperature for 15 min before microscopic observation. Ten microscopic fields were observed per CV-stained slide under 1000X magnification on a Motic® BA410 Elite microscope (Ted Pella Inc., CA, USA).

### SCLM of strong and weak biofilms, and enzymatic profiling of planktonic and sessile cells

Glass slides with 4-day-old biofilms were dipped (3X) in chilled sterile milli-Q water and stained with the LIVE/DEAD BacLight^*TM*^ Bacterial Viability kit (Invitrogen, Molecular Probes, Eugene, Oregon, USA) to determine the distribution of living and dead or inactive cells in biofilm. A drop of mounting antifade oil (Invitrogen) and a coverslip were placed on LIVE/DEAD-stained slides for immediate microscopic observation, or alternatively, stored at 4°C in the dark until observation and imaging (but no more than 30 min). Planktonic cells collected from supernatants (300 μL) surrounding 4-day-old biofilms were also stained with the LIVE/DEAD kit following the manufacturer's recommendations, and 10 μL were then placed on sterile slides for mounting in the dark as previously described. LIVE/DEAD-stained samples were observed through a Leica TCS SP5 II spectral confocal microscope (Leica Microsystems Inc., Jena, Germany), and the images were collected using the HCX PL APO 100x/1.44 Oil CORR CS objective and Leica Confocal software (version 2.6, Leica Inc.). The biofilm volume and percentage of living and dead cells were quantified from collected images using the ImageJ software package (Collins, 2007). Cells with yellow-green and orange-red emissions were considered as living and dead (or inactive) bacteria, respectively. Pearson's correlation coefficients between (1) expected proportions of live:dead bacteria (i.e., 0:100, 10:90, 50:50, 90:10, and 100:0) in cell suspensions prepared in accordance with the manufacturer's instructions (except that dead cells were prepared by an overnight formaldehyde-fixation, 10% v/v), and (2) empirical proportions of live: dead cells in the respective suspensions as determined by microscopic counting, indicated that the LIVE/DEAD kit is consistent to detect the contrasting physiological states in *F. psychrophilum* LM-02-Fp (*r* = 0.9966, *P* < 0.005) and NCMB1947^T^ (*r* = 0.9969, *P* < 0.005).

To detect specific components of the exopolysaccharide (EPS) matrix and embedded cells in mature biofilms, 4-day-old biofilms were fixed in chilled methanol for 15 min, dipped (1X) in sterile milli-Q water, air-dried at room temperature, and stained with fluorophore-conjugated lectins and DAPI (4′,6-diamidino-2-phenylindole, Sigma-Aldrich) in accordance with the providers' specifications. The lectins used were FITC-conjugated concanavalin A (ConA, 100 μg mL^−1^; Sigma-Aldrich) and Alexa fluor 488-conjugated wheat germ agglutinin (WGA, 5 μg mL^−1^; Invitrogen). A drop of mounting medium (Dako, Invitrogen) and a coverslip were placed on fluor-stained slides for subsequent observation and imaging of the EPS matrix and cells. The images were collected as previously described, and at least 10 microscopic fields were analyzed per sample.

The phenotypic features of 4-day-old biofilms and their respective planktonic cells were determined by enzymatic profiling with the miniaturized API ZYM® system in accordance with the manufacturer's directions (API-BioMerieux, La Balme-les-Grottes, France). To do so, 6 and 12 glass slides were collected from each experiment of strong and weak biofilm formation, respectively, washed (3X) by dipping in chilled sterile milli-Q water, and then scraped for cell detachment with sterile cell scrapers. Detached cells from strong and weak biofilms were separately concentrated in 1 mL of chilled sterile milli-Q water, and turbidity was adjusted to 3.0 McFarland for API ZYM assays. The efficiency of cell detachment was checked by microscopic observation of scraped slides that were stained with a CV solution (1% w/v), and compared with CV-stained slides from the negative controls. In addition, free-living cells in supernatants of strong and weak biofilms were harvested by centrifugation (7,800 rpm) at 4°C for 2.5 min. The resultant cell pellets were washed (1X) with chilled sterile milli-Q water and then separately resuspended in 1 mL of the same solvent at a density of 3.0 McFarland for API ZYM assays.

### Extraction of total RNA and RT-qpcr

RNA isolation from planktonic and biofilm samples of the LM-02-Fp and NCMB1947^T^ strains was done with the TRIzol® Max™ Bacterial RNA Isolation Kit (Ambion, Thermo Fisher Scientific, NY, USA). Planktonic cells in 4-day-old biofilm supernatants and 2-day-old cultures (1 mL) were harvested by centrifugation (7,800 rpm) at 4°C for 2.5 min, and each pellet of cells was homogenized and lysed with 1 mL of TRIzol reagent, according to the manufacturer's specifications. On the other hand, glass slides with 4-day-old biofilms were collected and washed (3X) by dipping into chilled sterile milli-Q water to discard non-adherent cells. Washed slides were transferred to a new 6-well microplate and 1 mL of TRIzol reagent was added per well for biofilm detachment (using a cell scraper) and cellular lysis. Twelve glass slides were processed with TRIzol in each biofilm formation experiment, and the corresponding aqueous phases (containing the RNA) were pooled in the isopropanol precipitation step to get a single extract of biofilm RNA per strain. A cell detachment efficiency of 100% was determined by microscopic observation of TRIzol-processed slides that were randomly chosen and stained with a CV solution (1% w/v). Concentration and quality (A_260_/A_280_ ratio) of total RNA preparations were determined using a Nanodrop® ND-1000 spectrophotometer (Thermo Fisher Scientific). All formaldehyde-agarose gel electrophoresis were performed as described by Rosen and Villa-Komaroff (1990), including a ssRNA ladder in each electrophoresis (0.5–9.0 Kb; New England. Biolabs, MA, USA).

The synthesis of complementary DNA (cDNA) was done with the ImProm-II™ Reverse Transcription System using 100 ng of DNase-treated RNA (TURBO DNA-free™ kit, Applied Biosystems, Austin, TX, USA) and reverse primers of six primer sets (Supplementary Table 1) designed for RT-qPCR of genes encoding putative virulence factors. The locus tags of these genes in *F. psychrophilum* strain JIP02/86 are as follows: FP0063, FP0097, FP0232, FP0595, FP1830, and FP2019. Primer sets were designed using the software Primer3 (version 4.0.0, Rozen and Skaletsky, 2000) and Beacon Designer™ (free edition) to check for primer secondary structures. The specificity of these primers was confirmed by cloning randomly selected amplicons using the pGEM®-T Easy Vector System (Promega, Madison, CA, USA), and the sequencing was carried out by Macrogen Inc. (Seoul, Korea). The clone sequences were stored in the GenBank public database with the access numbers KX600517 to KX600523. In addition, 16S rRNA gene transcripts of *F. psychrophilum* were quantified by RT-qPCR with the specific primer set Fp_16S1_fw (5′-GAGTTGGCATCAACACAC-3′) and Fp_16Sint1_rev (5′-TCCGTGTCTCAGTACCAG-3′) (Orieux et al., 2011).

All RT-qPCR were carried out on a volume of 16 μL containing 1 μL of cDNA, 2X Brilliant II SYBR® Green QPCR Master Mix (1X final concentration; Agilent Technologies-Stratagene, La Jolla, CA, USA), forward and reverse primers (1 μM, final concentration; Supplementary Table 1), and ROX as a passive reference dye (20 nM, final concentration; Agilent Technologies). The qPCR program consisted of the following steps: initial denaturation (3 min at 95°C), then 40 amplification cycles (30 s at 95°C), followed by 45 s annealing at 52°C (for FP0097, FP0232, and FP1830 genes) or 55°C (for FP0063, FP0595, FP2019, and 16S rRNA genes), and 45 s extension at 72°C. Clone sequences obtained in this study (KX600517 to KX600523) were used for absolute gene quantification by preparing 7-point standard curves (in triplicate) via 10-fold dilution series from 4 × 10^6^ copies. The efficiencies (E_%_) and correlation coefficients (*r*^2^) for standard curves were: FP0063 (E_%_ = 95.4–98.8, *r*^2^ = 0.993–0.998), FP0097 (E_%_ = 88.1–97.5, *r*^2^ = 0.920–0.993), FP0232 (E_%_ = 84.5–92.5, *r*^2^ = 0.808–0.996), FP0595 (E_%_ = 94.4–96.5, *r*^2^ = 0.990–0.999), FP1830 (E_%_ = 92.7–93.4, *r*^2^ = 0.998–0.999), FP2019 (E_%_ = 99.1–99.8, *r*^2^ = 0.921–0.957), and 16S rRNA gene (E_%_ = 93.7–96.1, *r*^2^ = 0.898–0.992). All RT-qPCR assays were performed using a Stratagene Mx3000P real-time PCR device (Agilent Technologies-Stratagene) and the data were analyzed using the software MXPro (version 4.10; Agilent Technologies).

### RNA-Seq and data processing

Next-generation sequencing libraries were prepared in the AUSTRAL-omics Laboratory (Universidad Austral de Chile) starting from total RNA samples extracted from the LM-02-Fp and NCMB1947^T^ strains grown under biofilm (on glass slides) and planktonic conditions as previously described. Each of these samples corresponded to a mixture of total RNA samples derived from four independent experiments that were mixed at the same concentration. RIN scores of the total RNA samples were determined on an Agilent Bioanalyzer using the RNA 6,000 Nano kit in accordance with the manufacturer's instructions (Agilent Technologies, CA, USA). We tried different hybridization-based kits for rRNA depleting in the total RNA samples, namely: RiboMinus™ Transcriptome Isolation Kit (for Yeast and bacteria; Invitrogen, CA, USA), Ribo-Zero™ rRNA Removal Kit (for bacteria; Epicentre, WI, USA) and MICROBExpress™ Bacterial mRNA Enrichment Kit (Ambion, Thermo Fisher Scientific, NY, USA). However, efficient depletion was achieved only when hybridization probes from the three kits were combined and used in two-rounds of depletion as part of the MICROBExpress™ Kit protocol. In addition, ssRNA (<100 nt) and dsRNA (<200 bp) were removed using the MEGAclear™ Kit (Ambion) in accordance with the manufacturer's instructions. Agilent Bioanalyzer profiling of the depleted samples confirmed the efficiency of the rRNA depletion procedure. Briefly, the libraries were generated using the KAPA Stranded mRNA-Seq Kit for Illumina platforms following the manufacturer's instructions (Kapa Biosystems Inc., MA, USA), and each library was tagged with a specific barcode that allowed sequencing of pooled libraries. The average size of the library fragments was determined by running the libraries on a Fragment Analyzer™ system (Advanced Analytical Technologies, IA, USA) and using the DNF-479 Standard Sensitivity NGS Fragment Analysis Kit. Library concentrations were determined on a LightCycler® Nano Real-Time PCR instrument (Roche Diagnostics, GmbH) using the KAPA Library Quantification Kit for Illumina platforms (Kapa Biosystems, Inc.) in order to normalize their concentrations at 10 nM. Libraries were pooled in an equimolar ratio using a protocol for denaturing and diluting libraries from MiSeq System, and then loaded (at 12 pM with 1% PhiX, final concentration) on an Illumina MiSeq instrument (Illumina, San Diego, CA, USA) to generate paired-end reads using the v2 MiSeq chemistry with the longest read length set at 2 × 150 bp, according to the manufacturer's instructions. RNA-Seq data were deposited in the European Nucleotide Archive (ENA) under study number PRJEB14670 for planktonic (ERS1231641, ERS1231642) and biofilm samples (ERS1231643, ERS1231644).

Technical sequences (e.g., adapters) were removed and quality trimming was done using Trimmomatic (Bolger et al., 2014) and PRINSEQ (Schmieder and Edwards, 2011), respectively, and discarding bases (or sequences) with Phred quality scores lower than 30. Genes were extracted from a reference genome, *F. psychrophilum* strain JIP02/86 (NCBI accession number NC_009613.3), using the “gffread” option in BedTools (Quinlan and Hall, 2010) to generate a transcript fasta file with the coordinates of all genes in the genome. The high-quality reads were mapped to these genes using the Burrows-Wheeler Alignment tool (Li and Durbin, 2009), which creates “.bam” output files. The number of sequence reads per gene for each “.bam” file was computed using the “multiBamCov” option in BedTools and the transcript fasta file. The functional annotation of high-quality reads was done using Blast2GO software (Conesa et al., 2005) based on the BLAST algorithm and GO annotations for the hit sequences.

### Data analysis

RT-qPCR data were normalized by the total RNA concentration of a given sample and log_2_ transformed for making comparisons. Normalized RT-qPCR counts were also used to compute the log_2_-fold change (-FC) ratio between biofilm and planktonic states. The lower and upper thresholds for changes were equal to 4 (i.e., log_2_-FC ratio ≥ 2) and 0.25 folds (i.e., log_2_-FC ratio ≤ −2) for upregulated and downregulated genes in the biofilm state, respectively.

High-quality read sequences were total count (TC) normalized (Dillies et al., 2012) and log_2_ transformed to visualize the global changes in gene expression by the heatmap.2 function in the R's gplots package (Version 3.0.1; Warnes et al., 2013)^3^. Cluster analyses were performed using the Manhattan index of distance and UPGMA linkage clustering. The columns were Z-score scaled for visualization purposes. To evaluate confidence in the clustering results, an approximately unbiased (AU) *P*-value was computed after re-sampling 10,000 times. The AU *P*-value is less biased than the bootstrap probability value computed by ordinary bootstrapping (version 2.0-0; Suzuki and Shimodaira, 2006)^4^. In addition, differentially expressed genes (DEGs) between the biofilm and planktonic states were identified using the DESeq2 package (Love et al., 2014). To do so, read counts from the biofilm and planktonic states were separately normalized using TMM normalization of the edgeR Bioconductor package (Robinson et al., 2010), and then simultaneously processed with DEseq2. The lower and upper thresholds for changes were also 4 (log_2_-FC ≥ 2) and 0.25 folds (log_2_-FC ≤ −2) for upregulated and downregulated genes in the biofilm state, respectively, and with *P*adj-values of 0.001 or less.

## Results

### *Flavobacterium psychrophilum* biofilm development

**Figure 1:**
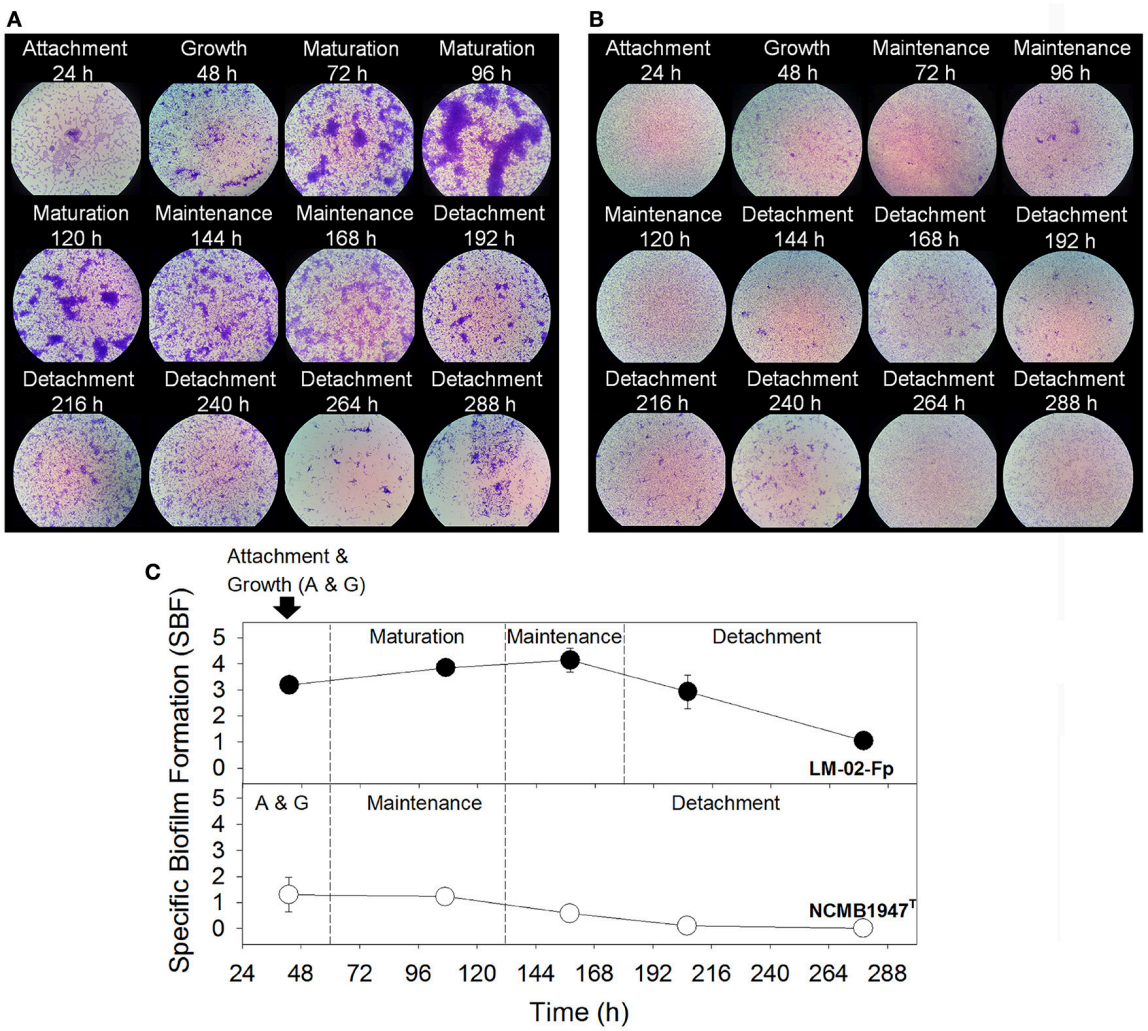
*****F. psychrophilum*** biofilm production. (A)** Strong and **(B)** weak biofilms produced by the LM-02-Fp and NCMB1947^T^ strains, respectively (1000X magnification). **(C)** Different biofilm development stages as determined by the specific biofilm formation index. Results are representative of three independent experiments.

The biofilm analyses indicated that *F. psychrophilum* LM-02-Fp and NCMB1947^T^ were, respectively, the strongest and weakest biofilm producers among the evaluated strains (Supplementary Figure 1). Overall, biofilm formation tended to diminish toward 96 h for all strains, except for strain LM-02-Fp that showed the opposite tendency (Supplementary Figure 1). The LM-02-Fp biofilms were characterized by initial cell attachment and microcolony formation in the course of the first 48 h, and visualization of the first cellular aggregates at 72 h (Figure 1A). This strain showed maximum biofilm formation between 96 and 120 h (mature biofilm), including a maintenance period between ca. 144 and 168 h that was followed by cell detachment (Figures 1A,C). Cell detachment was significant but not total after 192 h and adherent cells were still detectable at 268 h (Figures 1A,C). The NCMB1947^T^ biofilms followed a different development pattern of the strong-biofilm producer. The initial adherence and growth of the NCMB1947^T^ strain (within the first 48 h) were immediately followed by a maintenance period, without an apparent maturation stage (Figures 1B,C), and with only a few microcolonies detected during the entire study period (Figure 1B). Cell detachment started at approximately 144 h, being significant (but slow and not complete) after 168 h (Figures 1B,C).

**Figure 2:**
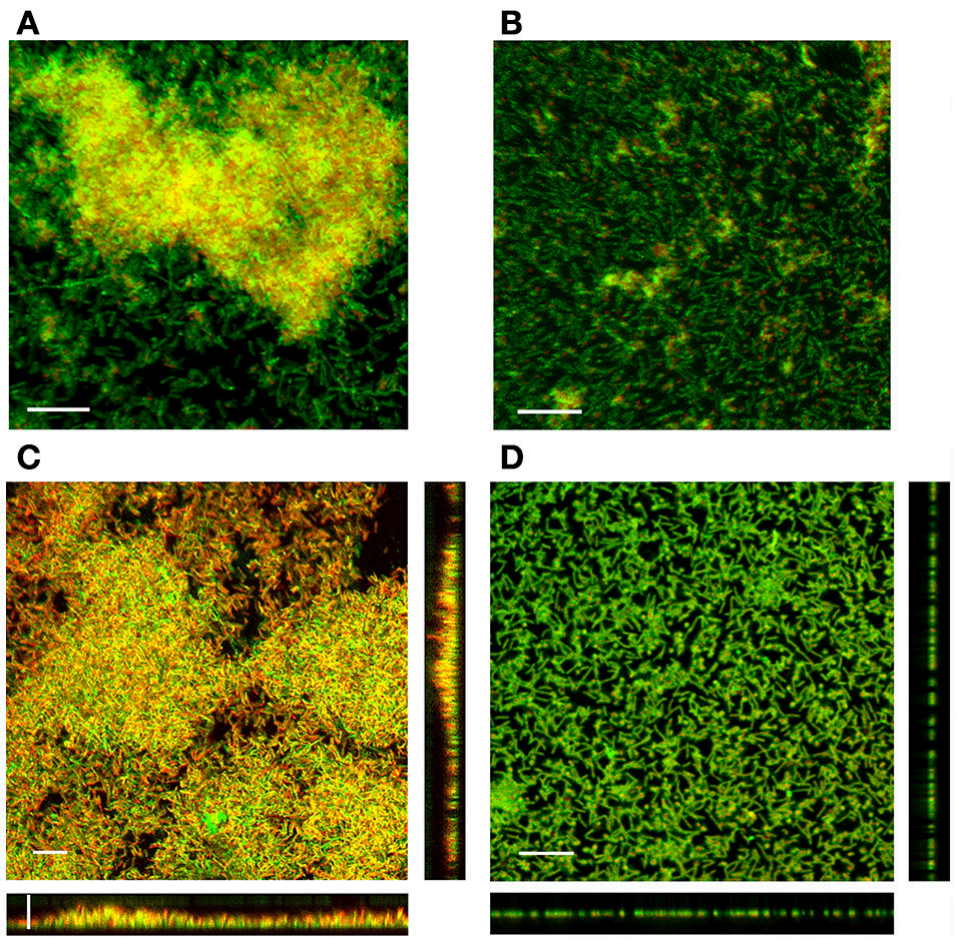
**SCLM micrographs displaying the EPS matrix and 3D-structure of 4-day-old biofilms**. The EPS matrix in **(A)** LM-02-Fp and **(B)** NCMB1947^T^ biofilms as detected by WGA lectin. The embedded cells in the EPS matrix were stained with DAPI staining and depicted in orange solely for visualization purposes. **(C)** LM-02-Fp and **(D)** NCMB1947^T^ biofilms stained with LIVE/DEAD reagent. The scale bar on the micrographs denotes a size of 10 μm. Results are representative of three independent experiments.

Four-day-old biofilms formed by the LM-02-Fp strain consisted of cell multilayers that were embedded in a three-dimensional EPS matrix composed of *N*-acetyl sugars as suggested by staining with fluor-conjugated WGA (Figure 2A). However, the EPS signature was not detectable with FITC-conjugated ConA. The EPS signature was also detectable with fluor-conjugated WGA (but not with FITC-conjugated ConA) in NCMB1947^T^ biofilms at 96 h (Figure 2B).

### SCLM of mature biofilms and enzymatic profiles

Four-day-old biofilms of *F. psychrophilum* LM-02-Fp had an average thickness of 7 μm, with living cells found mostly in the deeper and intermediate layers of biofilms, and dead (and/or inactive) cells in the upper layers (Figure 2C). Image analyses indicated that living cells accounted for 43 ± 7% (±SD) of the total biofilm cells whereas the remainder cells were inactive and/or dead. Higher and variable percentages of living cells (56 ± 28%) were determined by LIVE/DEAD staining from detached cell samples at 96 h. In addition, living cells accounted for 79 ± 11% of the planktonic cells on LM-02-Fp biofilms at 96 h, and their culturability on TYES agar plates was not lost before 222 ± 23 h, although their concentrations (as a CFU) were decreased by at least one order of magnitude as compared with the initial inocula after 48 h incubation (data not shown). The LM-02-Fp strain showed 5 enzymatic activities (alkaline phosphatase, esterase C4, leucine arylamidase, acid phosphatase, and naphthol phosphohydrolase) that were clearly detectable in biofilm and planktonic states at 96 h, although only esterase and acid phosphatase activities showed differences between states, with their corresponding colorimetric reactions being more intense in biofilm cells (data not shown).

Four-day-old biofilms of the NCMB1947^T^ strain were mostly composed of living cells (91 ± 2.8%) as revealed by image analyses (Figure 2D), although a lower and variable percentage of living cells (78 ± 28%) was determined by LIVE/DEAD staining from detached cell samples at 96 h. These biofilms were basically arranged in a cell monolayer (with a thickness of ca. 1 μm) on the glass slides (Figure 2D), and their respective supernatants also contained a significant fraction of living cells (80 ± 10%). The loss of culturability of planktonic cells surrounding the NCMB1947^T^ biofilms was observed at 147 ± 37 h, although their concentrations (as a CFU) were also at least one order of magnitude lower than the initial inocula after 48 h incubation (data not shown). The NCMB1947^T^strain showed the same 5 aforementioned enzymatic activities in the biofilm and planktonic states at 96 h, with similar detection intensities among states, excepting for esterase activity, which was more evident by colorimetric detection in biofilm cells (data not shown).

### Global changes in gene expression by RNA-seq and RT-qPCR contrasting

**Figure 3:**
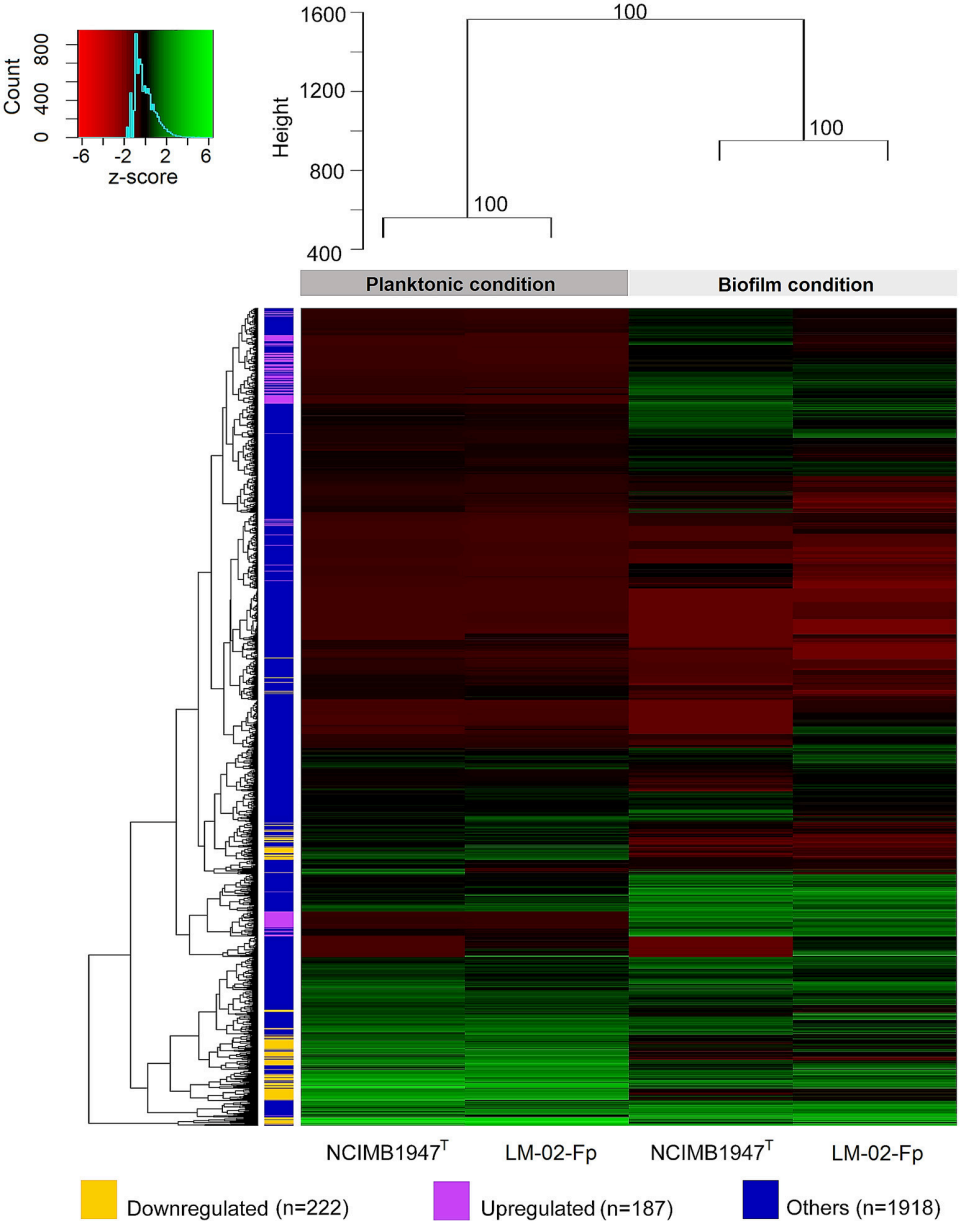
**Global shift patterns in gene expression of ***F. psychrophilum*** strains grown in biofilm and planktonic states**. Four-day-old biofilms were compared with free-living cells from 2-day-old cultures (see methodological considerations). The upper dendrogram shows strain clusters according to the growth phase (AU *P* > 90), while dendrogram on the left shows clusters encompassing genes with similar log_2_-TC normalized counts. The bulk fraction of genes, in blue (*n* = 1918 genes), did not show differential expression between the biofilm and planktonic states. This fraction includes (i) common genes (*n* = 1771) with log_2_-FC values between > −2 and <2, (ii) genes with log_2_-FC values ≥ 2 (*n* = 54) and ≤ −2 (*n* = 41) but with *P*adj-values > 0.001, and (iii) genes whose transcription was only detected in biofilm (*n* = 2) or planktonic state (*n* = 50). The remaining genes were significantly (*P*adj-value ≤ 0.001) downregulated (in yellow; *n* = 222, log_2_-FC ≤ −2) or upregulated (in purple; *n* = 187, log_2_-FC ≥ 2) in the biofilm state.

The transcriptomes of *F. psychrophilum* NCMB1947^T^ and LM-02-Fp were composed of high-quality reads for a total of 2327 genes in biofilm and planktonic states. Cluster analysis showed that the transcriptomes were significantly grouped by state rather than by strain (AU *P* > 90), indicating important differences among states instead of among strains (Figure 3). Thus, the fold-change (FC) of gene expression levels between states was computed regardless of the strain, and the effects of inter-strain variation were reduced by considering only genes with log_2_-FC values ≤ −2 and ≥ 2 and *P*adj-values ≤ 0.001 as DEGs (see Materials and Methods). A total of 222 and 187 genes were significantly downregulated (log_2_-FC ≤ −2) and upregulated (log_2_-FC ≥ 2), respectively, in the biofilm state (Figure 3). The remaining genes (*n* = 1918, i.e., ~82% of total genes in *F. psychrophilum* transcriptomes) were considered non-DEGs between biofilm and planktonic states (Figure 3).

**Figure 4:**
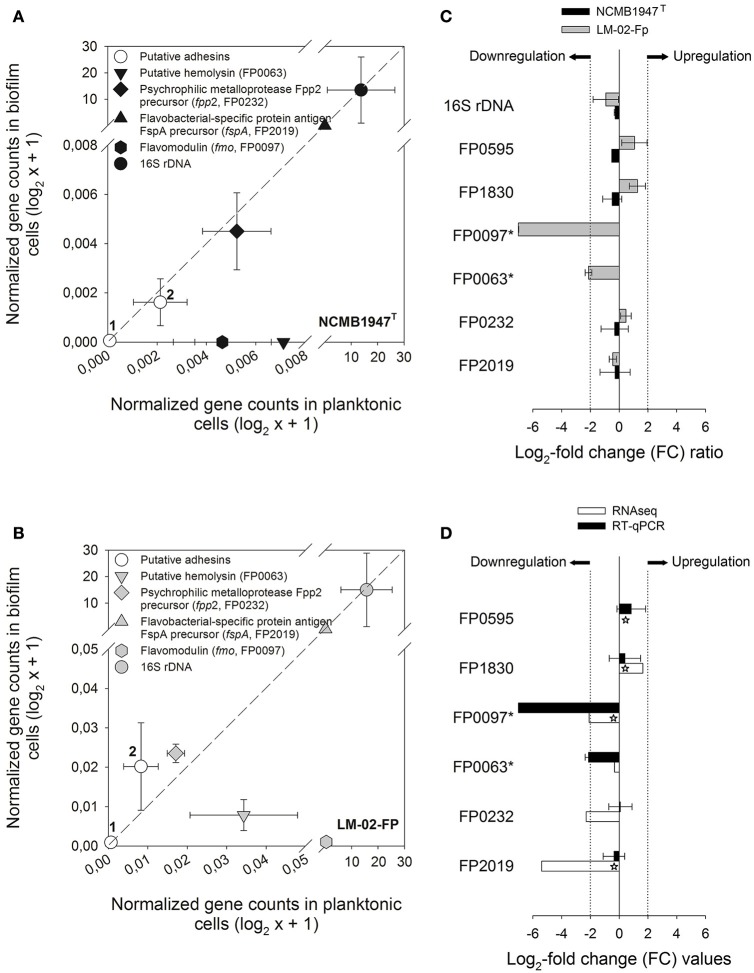
**Expression analysis of specific genes in biofilm and planktonic states**. RT-qPCR data from 4-day-old biofilms were compared against their respective planktonic counterparts for the **(A)** NCMB1947^T^ and **(B)** LM-02-Fp strains. Symbols represent averages from three independent experiments. Open circles indicate putative adhesins encoded by the FP0595 and FP1830 genes (1 and 2, respectively). The diagonal dashed lines indicate a perfectly linear relationship (1:1 ratio) between biofilm and planktonic RT-qPCR data. **(C)** Fold change (FC) ratios for specific genes computed from biofilm and planktonic RT-qPCR data. A FC value was not computable for asterisk-labeled genes in the NCMB1947^T^ strain as these were only detected in the planktonic state. **(D)** RT-qPCR- vs. RNA-Seq-based FC for specific genes in black and white bars, respectively. Significant FC values derived from RNA-Seq data are indicated by stars on white bars (*P*adj-value ≤ 0.001). Log_2_-FC values ≥ 2 and ≤ −2 in **(C,D)** indicate upregulated and downregulated genes in the biofilm state, respectively.

The transcriptional level of six specific genes encoding putative virulence factors was determined by RT-qPCR in 4-day-old biofilms and their corresponding supernatants (Figure 4). These assays revealed that five genes (including the 16S rRNA gene) were expressed in a ratio of nearly 1 to 1 between the biofilm and planktonic phases (Figures 4A,B), and therefore can be considered as non-DEGs, along with the FP0063 gene (Figure 4C). The FP0063 gene was undetectable in NCMB1947^T^ biofilms (Figure 4A) and slightly downregulated in LM-02-Fp biofilms compared with the FP0097 gene (Figures 4B,C). The gene transcription levels determined by RT-qPCR were expressed as a FC of expression levels between biofilm and planktonic states without distinction of the strain, and then compared with those estimated by RNA-Seq. The results indicated that the general trend in gene expression for specific genes was similar between RT-qPCR and RNA-Seq (Figure 4D).

### Virulence- and biofilm-related genes (VBRGs) in biofilm and planktonic states

**Figure 5:**
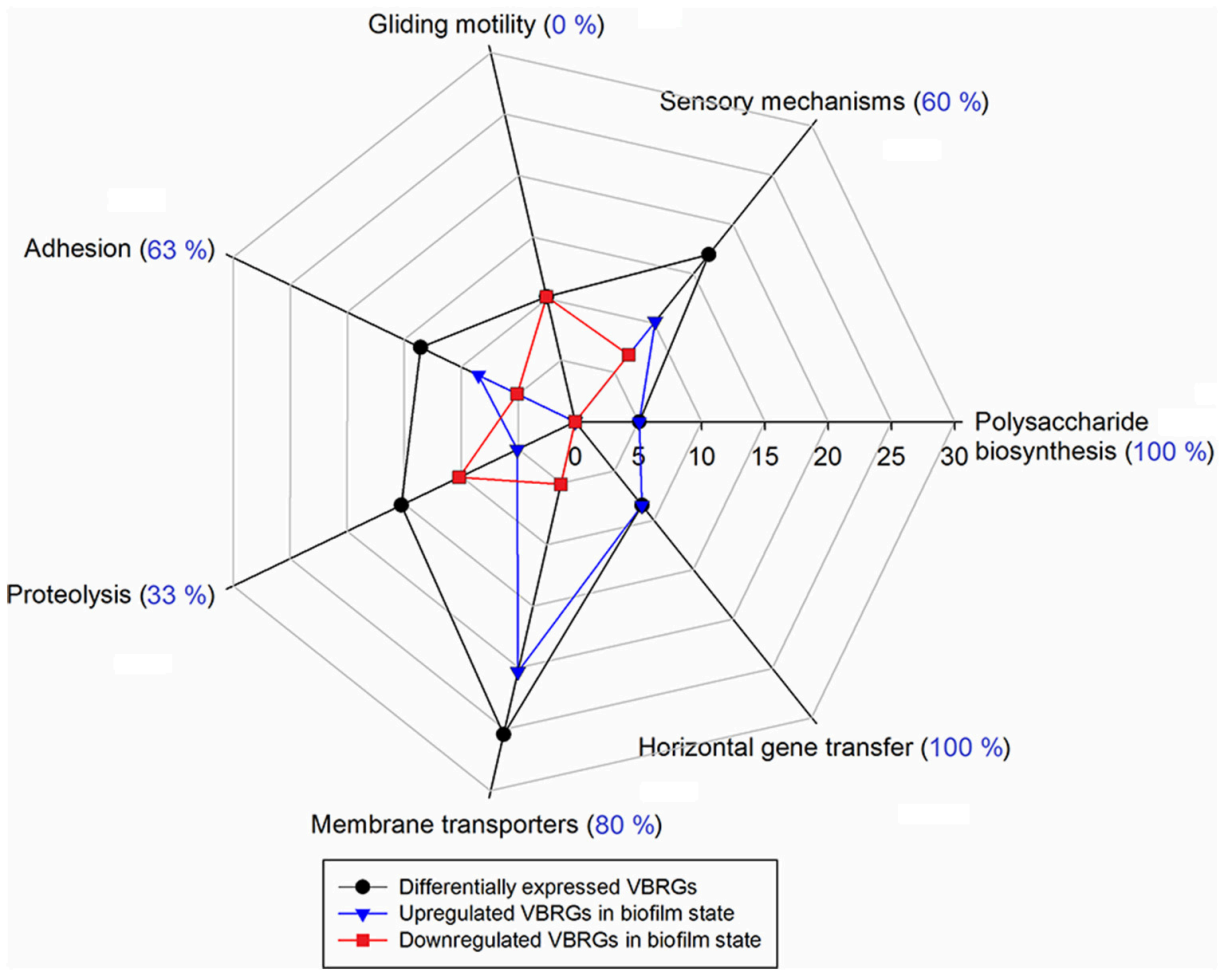
**Percentage of putative virulence- and biofilm-related genes (VBRGs) of ***F. psychrophilum*** LM-02-Fp and NCMB1947^**T**^ and biological function**. Differentially expressed VBRGs (in black) for a given biological function include upregulated (in blue) and downregulated genes (in red) in the biofilm state. These genes are expressed as a percentage of the total VBRGs (Table 1) along the axis that starts at the center of the plot. In addition, upregulated VBRGs in a given biological function are expressed as a percentage of differentially expressed VBRGs within the same functional category and shown in parentheses.

Differentially expressed VBRGs were associated with the polysaccharide biosynthesis, signal integration via a two-component system, motility, adhesion, proteolysis, membrane drug transport, and horizontal gene transfer. Particularly, VBRGs with roles in polysaccharide biosynthesis, horizontal gene transfer, adhesion, sensory mechanisms, and membrane transport, were all, or most of them, significantly upregulated in the biofilm state, and represented 5.1, 8.5, 13.6, 16.9, and 25.4% of the total number of VBRGs, respectively (Table 1 and Figure 5). Conversely, all motility-related genes were significantly downregulated in the biofilm state, and represented 10.2% of total VBRGs (Table 1 and Figure 5). Only about 33% of proteolysis-related genes was significantly upregulated in the biofilm state, and all of these represented 15.3% of total VBRGs (Table 1 and Figure 5). In addition, genes FP2536 and FP2534 encoding a transposase belonging to the ISNCY family and a putative transposase, respectively, are VBRGs that were only detected in the planktonic state.

**Table 1:** **Differentially expressed genes encoding possible virulence- and biofilm-related proteins in ***F. psychrophilum*** NCMB1947^**T**^ and LM-02-Fp**.

**Category**	**Gene symbol**	Locus tag^*^	**Description**	**Log_2_ FC (*P*adj-value ≤ 0.001)**
Polysaccharide biosynthesis proteins	FP1244	FP1244	Sugar transferase	2.27
	FP2036	FP2036	Probable transmembrane protein of unknown function (putative exosortase)	4.18
	FP2451	FP2451	Probable polysaccharide biosynthesis protein	2.78
Sensory mechanisms	*porX*	FP1066	Probable two-component system response regulatory protein containing PglZ domain PorX	−2.03
	FP1405	FP1405	Two-component system sensor histidine kinase	2.58
	FP1516	FP1516	Two-component system sensor histidine kinase	2.47
	FP1523	FP1523	Two-component system response regulatory protein	2.66
	FP1688	FP1688	Probable two-component system sensory protein	2.79
	FP1694	FP1694	Probable sigma-54 dependent two-component system response regulatory protein	2.21
	*rprY*	FP1826	Two-component system response regulatory protein RprY	−2.54
	FP1944	FP1944	LemA family protein (related to two-component system proteins)	−2.95
	FP2071	FP2071	Two-component system response regulatory protein, LuxR family	−2.06
	*porY*	FP2349	Two-component system sensor histidine kinase PorY	2.03
Motility	*remF*	FP0012	Gliding motility protein RemF precursor	−2.80
	*remG*	FP0013	Gliding motility protein RemG precursor	−3.94
	*gldJ*	FP1389	Gliding motility lipoprotein precursor GldJ	−2.64
	*gldN*	FP1970	Gliding motility protein precursor GldN	−3.58
	*gldM*	FP1971	Gliding motility transmembrane protein GldM	−3.45
	*gldL*	FP1972	Gliding motility transmembrane protein GldL	−3.58
Putative adhesins and adhesion-related proteins	*sprC*	FP0014	Putative adhesin precursor SprC	−2.29
	FP0139	FP0139	Probable outer membrane protein precursor, OmpA family	−3.30
	FP0156	FP0156	Outer membrane protein precursor; OmpA family P60	−5.22
	FP0546	FP0546	Probable outer membrane protein precursor, OmpA family	2.37
	FP1499	FP1499	Protein of unknown function precursor; putative adhesin	2.44
	FP1661	FP1661	Protein of unknown function precursor; putative adhesin	2.16
	FP1662	FP1662	Protein of unknown function precursor; putative adhesin	2.26
	FP2244	FP2244	Protein of unknown function precursor; putative adhesin	3.42
Proteolysis	FP0463	FP0463	Probable membrane associated S41A family C-terminal processing peptidase	2.78
	FP0886	FP0886	Probable S41 family peptidase precursor	2.04
	FP1003	FP1003	Probable S41 family peptidase	2.36
	*clpS*	FP1679	ATP-dependent Clp protease adaptor protein ClpS	−2.32
	*clpX*	FP2084	S14 family, ATP-dependent Clp protease ATP-binding subunit	−2.64
	*clpP*	FP2085	S14 family, ATP-dependent Clp protease proteolytic subunit	−2.02
	*clpA*	FP0747	ATPase with chaperone activity ATP-binding subunit (Clp protease component)	−2.08
	*lon*	FP1714	S16 family, ATP-dependent endopeptidase La	−2.48
	*clpB*	FP1765	ATPase with chaperone activity ATP-binding subunit (Clp protease component)	−2.52
Membrane transport	FP0351	FP0351	Probable drug/metabolite-transporting permease	2.50
	FP0420	FP0420	Probable outer membrane efflux protein precursor	2.25
	FP0503	FP0503	Probable membrane fusion efflux protein	−2.76
	FP0504	FP0504	Probable outer membrane efflux protein precursor	−2.32
	*phuR*	FP0522	Probable TonB-dependent outer membrane hemin receptor precursor PhuR	3.11
	*fsr*	FP0573	Major facilitator superfamily (MFS) permease. Fosmidomycin resistance protein	2.52
	*norM*	FP0569	Multidrug resistance protein norM (Na^+^/drug antiporter)	2.59
	FP0932	FP0932	Probable multidrug resistance protein. AcrB/AcrD/AcrF family protein	3.25
	FP1463	FP1463	Probable ABC-type iron(III)-transport system, binding lipoprotein precursor component	2.31
	FP1464	FP1464	Probable ABC-type iron(III)-transport system, permease component	4.08
	FP1809	FP1809	Probable multidrug resistance protein. AcrB/AcrD/AcrF family protein	5.73
	FP1810	FP1810	Probable membrane fusion efflux lipoprotein precursor	5.49
	FP1811	FP1811	Probable outer membrane efflux protein precursor	4.95
	FP0645	FP0645	Probable TonB-dependent outer membrane siderophore receptor precursor	2.77
	*tonB*	FP2207	Protein TonB	−2.82
Horizontal gene transfer	FP1391	FP1391	Putative transposase	4.42
	*xerD*	FP2020	Tyrosine recombinase XerD	2.62
	FP2539	FP2539	Transposase ISL3 family	4.23
	FP2569	FP2569	Transposase IS1182 family	3.07
	FP2570	FP2570	Transposase IS1182 family	4.82
Others	*fmo*	FP0097	Flavomodulin	−2.08
	FP1731	FP1731	Esterase/lipase/thioesterase family protein precursor	5.34
	FP2173	FP2173	Putative virulence-associated protein E (VapE)	7.22

*A fold change (FC) value for a given gene will be positive or negative if the gene is upregulated or downregulated in the biofilm state, respectively. Note that upregulated and downregulated genes in the biofilm state are, in turn, downregulated and upregulated genes in the planktonic state, respectively*.

(*) *Locus tags according to the genome of F. psychrophilum strain JIP02/86*.

## Discussion

All *F. psychrophilum* strains showed a variable capacity to form biofilms on non-living surfaces (Supplementary Figure 1), with remarkable differences between the LM-02-Fp and NCMB1947^T^ strains. This ability was a strain-dependent trait as in the case of other species of *Flavobacterium* (Basson et al., 2008). The LM-02-Fp strain showed four biofilm formation phases on polystyrene and glass surfaces, with a mature biofilm stage (i.e., cell multilayers with maximum biomass) reached between 96 and 120 h (Figures 1A,C, 2C). Previous works have reported (based on analysis of one or a few points in time) significant levels of biofilm formation by *F. psychrophilum* after 4–5 days of incubation (Álvarez et al., 2006; De la Fuente et al., 2013; Castillo et al., 2015), including the formation of dense cell aggregates by the NCMB1947^T^ strain on polystyrene microplates (Sundell and Wiklund, 2011). In our case, the NCMB1947^T^ strain did not show a perceptible maturation stage (compared to the LM-02-Fp strain) with significant formation of microcolonies (Figures 1B,C), and only formed a monolayered biofilm (Figure 2D). Our results suggest the presence of significant amounts of *N*-acetyl sugars (mostly *N*-acetyl-D-glucosamine as suggested by genomic information; (Duchaud et al., 2007)) in the EPS matrix of *F. psychrophilum* biofilms (Figures 2A,B). The main source of *N*-acetyl sugar components in the EPS matrix may have been WGA-binding OmpA-family proteins (Merle et al., 2003; Duchaud et al., 2007), and that can trigger a humoral response in rainbow trout (Dumetz et al., 2007). In addition, upregulation of a MFS permease gene (FP0573) in the biofilm state, along with three genes encoding polysaccharide biosynthesis-related proteins (FP1244, FP2036, and FP245; Table 1), is suggestive of active secretion of polysaccharides through membrane for biofilm formation. In fact, membrane-associated MFS transporters have previously been linked to the transport and secretion of EPS components in *F. johnsoniae* (Flemming, 2010). Polysaccharides are key structural components in biofilms of *F. columnare* (Cai et al., 2013), *Vibrio salmonicida* (Hansen et al., 2014), *Piscirickettsia salmonis* (Marshall et al., 2012), among other bacterial fish pathogens, and contribute directly to the biofilm emergent properties (Flemming et al., 2016). However, the polysaccharide composition of EPS matrix in *F. psychrophilum* biofilms is so far unknown.

Our results also indicate that *F. psychrophilum* requires several putative adhesins to form biofilms, along with the overexpression of FP0546 gene that encodes a protein precursor belonging to the OmpA family. It is likely that the active protein product of the FP0546 gene and some other upregulated adhesins (Table 1) are WGA-binding glycoproteins with *N*-acetyl sugar modifications. Indeed, some glycoproteins and OmpA-family proteins may participate in modulation of the adherence and pathogenicity in *F. psychrophilum* (Dumetz et al., 2007, 2008) and other fish-pathogenic bacteria such as *F. columnare* (Laanto et al., 2014). In addition, two putative adhesins were encoded by non-DEGs (FP0595 and FP183; Figures 4C,D), suggesting that these proteins may have a similar adhesion function in biofilm and planktonic states. Only three genes encoding putative adhesins were significantly downregulated in the biofilm state, among them, the *sprC* gene (Table 1) associated with gliding motility and chemotaxis (Duchaud et al., 2007). This result contrasts with a study of differential expression using *F. johnsoniae* strains grown under different substratum (Flemming, 2010), where a *sprD*-like gene was upregulated in biofilm rather than agar surface-associated phase.

All genes related to gliding machinery were significantly downregulated in the mature biofilm state compared to the planktonic state (Table 1 and Figure 5). Studies based on *F. psychrophilum* mutants that lack gliding motility have demonstrated that the acquisition of a non-motile phenotype is not only associated with enhanced biofilm production, but also with loss of extracellular protease activity and other virulent properties (Álvarez et al., 2006; Pérez-Pascual et al., 2015). In turn, mutations in the genes encoding proteolytic enzymes, such as the *fpp2* gene, can cause a hyper-gliding phenotype in this bacterium (Pérez-Pascual et al., 2011). In the present study, the FP0231 and FP0232 genes, which encode two of the better characterized extracellular proteolytic enzymes (Fpp1 and Fpp2, respectively; Secades et al., 2001, 2003), were identified as non-differentially expressed by RNA-Seq and RT-qPCR (e.g., see FP0232 in Figures 4C,D). This agrees with an earlier argument that both proteolytic enzymes can also be involved in nutrition-like processes (Pérez-Pascual et al., 2011), which could explain why both genes (FP0231 and FP0232) had similar expressions in biofilm and planktonic states. Among DEGs were several related to intracellular enzymes with proteolytic function rather than extracellular proteases (Table 1). Genes related to ATP-dependent protease complexes such as Lon and Clp proteases were significantly downregulated in the biofilm state. Clp protease is nonessential for growth in most pathogens, but is necessary for virulence and replication during infection (Raju et al., 2012). On the other hand, genes FP0463, FP0886, and FP1003, which encode S41 endopeptidases, were significantly upregulated in the biofilm state (Table 1). It has been demonstrated that the protein product of the MMCAP2_0241 gene, an S41 endopeptidase, confers the proteolytic phenotype to one of the most virulent mycoplasma species (Allam et al., 2012), and although there is low sequence similarity between genes FP0463/FP0886/FP1003 and MMCAP2_0241 (52–55% identity), a role of the first three genes in the virulence of *F. psychrophilum* should not be discarded without empirical evidence.

Completely sequenced genomes of *F. psychrophilum* indicate the presence of quorum-sensing-related genes encoding LuxR-like proteins, but not LuxI homologs^2^. Nonetheless, it is well-known that LuxR “Solos” (i.e., LuxR-like receptors that lack a cognate LuxI-like synthase) can also respond to ligands other than AHLs (Venturi and Ahmer, 2015). We identified two *luxR* “Solos” in our transcriptomes, one of them being classified as non-DEG (FP0715) and another significantly downregulated in the biofilm state (FP2071, Table 1). In agreement with these results and earlier findings (Bruhn et al., 2005), AHL-like molecules in cell-free culture supernatants were not detected by GC-MS (Supplementary Figure 2). These results suggest that expression of the putative transcription factors encoded by *luxR* “Solos” are not directly involved in biofilm formation by *F. psychrophilum*. In addition, 10 two-component system (TCS) related genes were found to be differentially expressed (Table 1), six of which were significantly upregulated in the biofilm state, including the FP1516 gene, which has been related to the control of biofilm formation in *F. psychrophilum* (Hesami et al., 2011). TCSs seem to be common in many fish-pathogenic bacteria (Guijarro et al., 2015) and hence the development of mutants for TCS-related genes will provide valuable insights into their roles in extracellular signal transduction and biofilm formation. We suggest that plankton-to-biofilm transition, and vice versa, could be modulated by a molecular mechanism based on a two–component phosphorylation cascade triggered by environmental stimuli other than AHL-like autoinducers, for instance, nutrients like iron. It has been suggested that *F. psychrophilum* is deficient in biofilm formation under iron-limiting conditions (De la Fuente et al., 2013), which is consistent with the overexpression of gliding machinery genes under such conditions (LaFrentz et al., 2009; Pérez-Pascual et al., 2009), since motility and biofilm formation are antagonistic traits in this bacterium (Álvarez et al., 2006; this study). Interestingly, two genes encoding iron transport-related proteins were significantly upregulated in the mature biofilm state (FP1463 and FP1464, Table 1), being probably responsible for the acquisition of this element under our (iron-nonlimiting) conditions. In contrast, TonB-dependent receptors (e.g., siderophores, Table 1) appear to play a minor role in the active transport across the membrane under sessile and iron-nonlimiting conditions, since the *tonB* gene was significantly downregulated in the biofilm state (FP2207, Table 1). Nevertheless, this result should be interpreted with caution, as TonB activity has been shown to be associated with biofilm production in other bacteria based on *tonB* mutants (e.g., Abbas et al., 2007).

*Flavobacterium psychrophilum* can acquire virulent genes from phylogenetically unrelated bacteria. For instance, it has been proposed that gene *fmo*, which encodes a pore-forming toxin known as flavomodulin (downregulated in the biofilm state, Table 1), is product of a horizontal gene transfer event from a Gram-positive bacterium (Dumetz et al., 2008). Some virulent strains of *F. psychrophilum* (Castillo et al., 2016) possess genomic islands that carry genes associated with toxins, drug resistance, and putative virulence factors such as virulence-associated protein E (VapE). Here, a *vapE*-like gene was significantly upregulated in the biofilm state (FP2173, Table 1). In addition, we identified one integrase (tyrosine recombinase XerD), one putative transposase (FP1391), and three transposases (namely, a ISL3 transposase and two IS1182 transposases) whose respective coding genes were all significantly upregulated in the biofilm state (Table 1 and Figure 5). In contrast, a transposase belonging to the ISNCY family (FP2536) and other putative transposase (FP2534) were only detected in the planktonic state, suggesting that a different set of transposases modulate gene transposition in the biofilm vs. planktonic state. Our results support the idea that spatial organization of biofilms facilitates horizontal gene transference and drug resistance via cell-to-cell communication. In fact, the simultaneous upregulation of five genes encoding proteins with a function related to antimicrobial drug transport through membrane (i.e., FP0351, *fsr, norM*, FP0932 and FP1809 genes; Table 1) could explain the rapid development of drug resistance in *F. psychrophilum* biofilms reported in an earlier study (Sundell and Wiklund, 2011). This may also be associated with the high levels of resistance to oxytetracycline, florfenicol, and oxolinic acid existing among Chilean isolates of *F. psychrophilum* (Henríquez-Núñez et al., 2012). Therefore, *F. psychrophilum* biofilms may be related to bacterial persistence under stressful conditions, as well as to the recurring infections caused by continuous exposure of target fish.

We have presented evidence indicating that *F. psychrophilum* strains that produce different biofilm phenotypes deploy a global transcriptional response in the mature biofilm state that differs substantially from their planktonic counterparts, but not among different strains. It has been suggested that *F. psychrophilum* changes from a virulent less-adherent free-living state to a non-virulent biofilm state, and vice versa. Conversely, we found that different biofilm phenotypes produced by different strains share a genetic potential for virulence that is transcriptionally enhanced with respect to free-living cells. In this context, *F. psychrophilum* biofilms act as an effective reservoir of putative virulence factors, and therefore are a factor that could explain how this bacterium triggers sudden BCWD/RTFS outbreaks in salmonid farms that are not explained by lack of staff training and/or adequate management.

## Author contributions

HL and RA participated in the rationale and design of the study. HL conducted the experiments, interpreted the data, and wrote the manuscript. RA participated in the critical review of the manuscript.

## Funding

This study was funded by FONDECYT Postdoctoral project 3150505 (CONICYT, Chile). We acknowledge partial financial support from FONDAP 15110027 (CONICYT, Chile).

### Conflict of interest statement

The authors declare that the research was conducted in the absence of any commercial or financial relationships that could be construed as a potential conflict of interest.

## Footnotes

^1^https://colloque.inra.fr/flavobacterium2009_eng/Proceedings

^2^www.ncbi.nlm.nih.gov/genome/genomes/1589

^3^https://cran.r-project.org/web/packages/gplots/index.html

^4^https://cran.r-project.org/web/packages/pvclust/index.html

## References

[B1] Abbas A. Adams C. Scully N. Glennon J. O'Gara F. (2007). A role for TonB1 in biofilm formation and quorum sensing in Pseudomonas aeruginosa. FEMS Microbiol. Lett. 274, 269–278. 10.1111/j.1574-6968.2007.00845.x 17623027

[B2] Allam A. B. Brown M. B. Reyes L. (2012). Disruption of the S41 peptidase gene in Mycoplasma mycoides capri impacts proteome profile, H2O2 production, and sensitivity to heat shock. PLoS ONE 7:e51345. 10.1371/journal.pone.0051345 23300541 PMC3534093

[B3] Álvarez B. Secades P. Prieto M. McBride M. J. Guijarro J. A. (2006). A mutation in *Flavobacterium psychrophilum* tlpB inhibits gliding motility and induces biofilm formation. Appl. Environ. Microbiol. 72, 4044–4453. 10.1128/AEM.00128-06 16751514 PMC1489658

[B4] Avendaño-Herrera R. Ilardi P. Fernández J. (2009). Significance of *Flavobacterium* diseases on salmonid farming in Chile, in 2nd Conference on Members of the Genus Flavobacterium (Paris). Available online at: https://colloque.inra.fr/flavobacterium2009_eng/Proceedings

[B5] Basson A. Flemming L. A. Chenia H. Y. (2008). Evaluation of adherence, hydrophobicity, aggregation, and biofilm development of Flavobacterium johnsoniae-like isolates. Microb. Ecol. 55, 1–14. 10.1007/s00248-007-9245-y 17401596

[B6] Bernardet J.-F. Bowman J. P. (2006). The genus *Flavobacterium*, in The Prokaryotes, eds Dworkin M. Falkow S. Rosenberg E. Schleifer K.-H. Stackebrandt E. (New York, NY: Springer-Verlag), 481–531.

[B7] Bernardet J.-F. Grimont P. A. D. (1989). Deoxyribonucleic acid relatedness and phenotypic characterization of *Flexibacter columnaris* sp. nov., nom. rev., *Flexibacter psychrophilus* sp. nov., nom. rev., and *Flexibacter maritimus* Wakabayashi, Hikida, and Masumura 1986. Int. J. Syst. Bacteriol. 39, 346–354. 10.1099/00207713-39-3-346

[B8] Bolger A. M. Lohse M. Usadel B. (2014). Trimmomatic: a flexible trimmer for Illumina sequence data. Bioinformatics. 30, 2114–2120. 10.1093/bioinformatics/btu170 24695404 PMC4103590

[B9] Bruhn J. B. Dalsgaard I. Nielsen K. F. Buchholtz C. Larsen J. L. Gram L. (2005). Quorum sensing signal molecules (acylated homoserine lactones) in gram-negative fish pathogenic bacteria. Dis. Aquat. Organ. 65, 43–52. 10.3354/dao065043 16042042

[B10] Cai W. De La Fuente L. Arias C. R. (2013). Biofilm formation by the fish pathogen *Flavobacterium columnare*: development and parameters affecting surface attachment. Appl. Environ. Microbiol. 79, 5633–5642. 10.1128/AEM.01192-13 23851087 PMC3754160

[B11] Castillo D. Christiansen R. H. Dalsgaard I. Madsen L. Espejo R. Middelboe M. (2016). Comparative genome analysis provides insights into the pathogenicity of *Flavobacterium psychrophilum*. PLoS ONE 11:e0152515. 10.1371/journal.pone.0152515 27071075 PMC4829187

[B12] Castillo D. Christiansen R. H. Dalsgaard I. Madsen L. Middelboe M. (2015). Bacteriophage resistance mechanisms in the fish pathogen *Flavobacterium psychrophilum*: linking genomic mutations to changes in bacterial virulence factors. Appl. Environ. Microbiol. 81, 1157–1167. 10.1128/AEM.03699-14 25480749 PMC4292493

[B13] Collins T. J. (2007). ImageJ for microscopy. Biotechniques. 43, 25–30. 10.2144/000112517 17936939

[B14] Conesa A. Götz S. García-Gómez J. M. Terol J. Talón M. Robles M. (2005). Blast2GO: a universal tool for annotation, visualization and analysis in functional genomics research. Bioinformatics 21, 3674–3676. 10.1093/bioinformatics/bti610 16081474

[B15] Davis H. S. (1946). Care and Diseases of Trout. United States Fish and Wildlife Service Research Report no. 12, Washington, DC: US Government Printing Office.

[B16] De la Fuente M. Vidal J. M. Miranda C. D. González G. Urrutia H. (2013). Inhibition of *Flavobacterium psychrophilum* biofilm formation using a biofilm of the antagonist *Pseudomonas fluorescens* FF48. Springerplus 2:176. 10.1186/2193-1801-2-176 23667820 PMC3650236

[B17] Dillies M.-A. Rau A. Aubert J. Hennequet-Antier C. Jeanmougin M. Servant N.. (2012). A comprehensive evaluation of normalization methods for Illumina high-throughput RNA sequencing data analysis. Brief. Bioinform. 14, 671–683. 10.1093/bib/bbs046 22988256

[B18] Duchaud E. Boussaha M. Loux V. Bernardet J. F. Michel C. Kerouault B.. (2007). Complete genome sequence of the fish pathogen *Flavobacterium psychrophilum*. Nat. Biotechnol. 25, 763–769. 10.1038/nbt1313 17592475

[B19] Dumetz F. Duchaud E. Claverol S. Orieux N. Papillon S. Lapaillerie D.. (2008). Analysis of the *Flavobacterium psychrophilum* outer-membrane subproteome and identification of new antigenic targets for vaccine by immunomics. Microbiology 154, 1793–1801. 10.1099/mic.0.2008/016600-0 18524934

[B20] Dumetz F. LaPatra S. E. Duchaud E. Claverol S. Le Henaff M. (2007). The *Flavobacterium psychrophilum* OmpA, an outer membrane glycoprotein, induces a humoral response in rainbow trout. J. Appl. Microbiol. 103, 1461–1470. 10.1111/j.1365-2672.2007.03359.x 17953557

[B21] Elsayed E. E. Eissa A. E. Faisal M. (2006). Isolation of *Flavobacterium psychrophilum* from sea lamprey, *Petromyzon marinus* L., with skin lesions in Lake Ontario. J. Fish Dis. 29, 629–632. 10.1111/j.1365-2761.2006.00756.x 17026672

[B22] Evensen O. Lorenzen E. (1997). Simultaneous demonstration of infectious pancreatic necrosis virus (IPNV) and *Flavobacterium psychrophilum* in paraffin-embedded specimens of rainbow trout *Oncorhynchus mykiss* fry by use of paired immunohistochemistry. Dis. Aquat. Organ. 29, 227–232. 10.3354/dao029227

[B23] Flemming H.-C. Wingender J. Kjelleberg S. Steinberg P. D. Rice S. A. Szewzyk U. (2016). Biofilms: an emergent form of bacterial life. Nat. Rev. Microbiol. 14, 563–575. 10.1038/nrmicro.2016.94 27510863

[B24] Flemming L. (2010). Comparative Proteomic and Genomic Analysis of Flavobacterium Johnsoniae-Like Biofilm, Planktonic and Agar Surface Associated Cells. Dissertation/PhD's thesis, Stellenbosch University, Stellenbosch.

[B25] Guijarro J. A. Cascales D. García-Torrico A. I. García-Domínguez M. Méndez J. (2015). Temperature-dependent expression of virulence genes in fish-pathogenic bacteria. Front. Microbiol. 6:700. 10.3389/fmicb.2015.00700 26217329 PMC4496569

[B26] Hansen H. Bjelland A. M. Ronessen M. Robertsen E. Willassen N. P. (2014). LitR is a repressor of syp genes and has a temperature-sensitive regulatory effect on biofilm formation and colony morphology in *Vibrio (Aliivibrio) salmonicida*. Appl. Environ. Microbiol. 80, 5530–5541. 10.1128/AEM.01239-14 24973072 PMC4136084

[B27] Henríquez-Núñez H. Evrard O. Kronvall G. Avenda-o-Herrera R. (2012). Antimicrobial susceptibility and plasmid profiles of *Flavobacterium psychrophilum* strains isolated in Chile. Aquaculture. 354–355, 38–44. 10.1016/j.aquaculture.2012.04.034

[B28] Hesami S. Metcalf D. S. Lumsden J. S. MacInnes J. I. (2011). Identification of cold-temperature-regulated genes in *Flavobacterium psychrophilum*. Appl. Environ. Microbiol. 77, 1593–1600. 10.1128/AEM.01717-10 21216906 PMC3067270

[B29] Högfors-Rönnholm E. Norrgård J. Wiklund T. (2014). Adhesion of smooth and rough phenotypes of *Flavobacterium psychrophilum* to polystyrene surfaces. J. Fish Dis. 38, 429–437. 10.1111/jfd.12250 24716830

[B30] Holt R. A. Rohovec J. S. Fryer J. L. (1993). Bacterial coldwater disease, in Bacterial Diseases of Fish, eds Inglis V. Roberts R. J. Bromage N. R. (Oxford: Blackwell Scientific Publications), 3–23.

[B31] Kastbjerg V. G. Nielsen K. F. Dalsgaard I. Rasch M. Bruhn J. B. Givskov M.. (2007). Profiling acylated homoserine lactones in *Yersinia ruckeri* and influence of exogenous acyl homoserine lactones and known quorum-sensing inhibitors on protease production. J. Appl. Microbiol. 102, 363–374. 10.1111/j.1365-2672.2006.03109.x 17241341

[B32] Kondo M. Kawai K. Kurohara K. Oshima S. (2002). Adherence of *Flavobacterium psychrophilum* on the body surface of the ayu Plecoglossus altivelis. Microb. Infect. 4, 279–283. 10.1016/S1286-4579(02)01539-3 11909737

[B33] Laanto E. Penttinen R. K. Bamford J. K. H. Sundberg L.-R. (2014). Comparing the different morphotypes of a fish pathogen-implications for key virulence factors in Flavobacterium columnare. BMC Microbiol. 14:170. 10.1186/1471-2180-14-170 24964840 PMC4094633

[B34] LaFrentz B. R. LaPatra S. E. Call D. R. Wiens G. D. Cain K. D. (2009). Proteomic analysis of *Flavobacterium psychrophilum* cultured *in vivo* and in iron-limited media. Dis. Aquat. Organ. 87, 171–182. 10.3354/dao02122 20099411

[B35] Leyton A. Urrutia H. Vidal J. M. de la Fuente M. Alarcón M. Aroca G. (2015). Inhibitory activity of Antarctic bacteria Pseudomonas sp. M19B on the biofilm formation of *Flavobacterium psychrophilum* 19749. Rev. Biol. Mar. Oceanog. 50, 375–381. 10.4067/S0718-19572015000300016

[B36] Li H. Durbin R. (2009). Fast and accurate short read alignment with Burrows-Wheeler Transform. Bioinformatics 25, 1754–1760. 10.1093/bioinformatics/btp324 19451168 PMC2705234

[B37] Liu Y. Morley M. Brandimarto J. Hannenhalli S. Hu Y. Ashley E. A.. (2015). RNA-Seq identifies novel myocardial gene expression signatures of heart failure. Genomics 105, 83–89. 10.1016/j.ygeno.2014.12.002 25528681 PMC4684258

[B38] Lorenzen E. Dalsgaard I. Bernardet J. F. (1997). Characterization of isolates of *Flavobacterium psychrophilum* associated with coldwater disease or rainbow trout fry syndrome I: phenotypic and genomic studies. Dis. Aquat. Org. 31, 197–208. 10.3354/dao031197

[B39] Love M. I. Huber W. Anders S. (2014). Moderated estimation of fold change and dispersion for RNA-Seq data with DESeq2. Genome Biol. 15:550. 10.1186/s13059-014-0550-8 25516281 PMC4302049

[B40] Marshall S. H. Gómez F. A. Ramírez R. Nilo L. Henríquez V. (2012). Biofilm generation by *Piscirickettsia salmonis* under growth stress conditions: a putative *in vivo* survival/persistence strategy in marine environments. Res. Microbiol. 163, 557–566. 10.1016/j.resmic.2012.08.002 22910282

[B41] Merle C. Faure D. Urdaci M.-C. Le Hénaff M. (2003). Purification and characterization of a membrane glycoprotein from the fish pathogen *Flavobacterium psychrophilum*. J. Appl. Microbiol. 94, 1120–1127. 10.1046/j.1365-2672.2003.01946.x 12752822

[B42] Møller J. D. Larsen J. L. Madsen L. Dalsgaard I. (2003). Involvement of a sialic acid-binding lectin with hemagglutination and hydrophobicity of *Flavobacterium psychrophilum*. Appl. Environ. Microbiol. 69, 5275–5280. 10.1128/AEM.69.9.5275-5280.2003 12957914 PMC194956

[B43] Morgan A. L. Thompson K. D. Adams A. (2009). The secret life of *Flavobacterium* psychrophilum, in 2nd Conference on Members of the Genus Flavobacterium (Paris). Available online at: https://colloque.inra.fr/flavobacterium2009_eng/Proceedings

[B44] Nematollahi A. Decostere A. Pasmans F. Ducatelle R. Haesebrouck F. (2003b). Adhesion of high and low virulence *Flavobacterium psychrophilum* strains to isolated gill arches of rainbow trout *Oncorhynchus mykiss*. Dis. Aquat. Org. 55, 101–107. 10.3354/dao055101 12911057

[B45] Nematollahi A. Decostere A. Pasmans F. Haesebrouck F. (2003a). *Flavobacterium psychrophilum* infections in salmonid fish. J. Fish Dis. 26, 563–574. 10.1046/j.1365-2761.2003.00488.x 14653314

[B46] Niu C. Gilbert E. S. (2004). Colorimetric method for identifying plant essential oil components that affect biofilm formation and structure. Appl. Environ. Microbiol. 70, 6951–6956. 10.1128/AEM.70.12.6951-6956.2004 15574886 PMC535164

[B47] Orieux N. Bourdineaud J. Douet D. Daniel P. Le Hénaff M. (2011). Quantification of *Flavobacterium psychrophilum* in rainbow trout, *Oncorhynchus mykiss* (Walbaum), tissues by qPCR. J. Fish Dis. 34, 811–821. 10.1111/j.1365-2761.2011.01296.x 21988353

[B48] Papadopoulou A. Howell A. Wiklund T. (2015). Inhibition of *Flavobacterium psychrophilum* adhesion *in vitro*. FEMS Microbiol. Lett. 362, 1–9. 10.1093/femsle/fnv203 26500088

[B49] Pérez-Pascual D. Gomez E. Álvarez B. Mendez J. Reimundo P. Navais R.. (2011). Comparative analysis and mutation effects of fpp2-fpp1 tandem genes encoding proteolytic extracellular enzymes of *Flavobacterium psychrophilum*. Microbiology 157, 1196–1204. 10.1099/mic.0.046938-0 21292745

[B50] Pérez-Pascual D. Gomez E. Guijarro J. A. (2015). Lack of a type-2 glycosyltransferase in the fish pathogen *Flavobacterium psychrophilum* determines pleiotropic changes and loss of virulence. Vet. Res. 46, 1. 10.1186/s13567-014-0124-5 25582708 PMC4293000

[B51] Pérez-Pascual D. Menéndez A. Fernández L. Mendéz J. Reimundo P. Navais R.. (2009). Spreading versus biomass production by colonies of the fish pathogen *Flavobacterium psychrophilum*: role of nutrient concentration. Int. Microbiol. 12, 207–214. 10.2436/20.1501.01.100 20112225

[B52] Quinlan A. R. Hall I. M. (2010). BEDTools: a flexible suite of utilities for comparing genomic features. Bioinformatics 26, 841–842. 10.1093/bioinformatics/btq033 20110278 PMC2832824

[B53] Raju R. M. Goldberg A. L. Rubin E. J. (2012). Bacterial proteolytic complexes as therapeutic targets. Nat. Rev. Drug Discov. 11, 777–789. 10.1038/nrd3846 23023677

[B54] Ríos-Castillo A. G. (2014). Evaluación del Nivel de Contaminación de Superficies y la Eficacia de Productos Desinfectantes a corto y Largo Plazo: Nuevos Métodos. Dissertation/PhD's thesis, Universitat Autònoma de Barcelona, Barcelona.

[B55] Robinson M. D. McCarthy D. J. Smyth G. K. (2010). edgeR: a Bioconductor package for differential expression analysis of digital gene expression data. Bioinformatics 26, 139–140. 10.1093/bioinformatics/btp616 19910308 PMC2796818

[B56] Rosen K. M. Villa-Komaroff L. (1990). An alternative method for the visualization of RNA in formaldehyde-agarose gels. Focus 12, 23–24.

[B57] Rozen S. Skaletsky H. (2000). Primer3 on the WWW for general users and for biologist programmers, in Bioinformatics Methods and Protocols: Methods in Molecular Biology, eds Misener S. Krawetz S. A. (Totowa, NJ: Humana Press), 365–386. 10.1385/1-59259-192-2:365 10547847

[B58] Schmieder R. Edwards R. (2011). Quality control and preprocessing of metagenomic datasets. Bioinformatics 27, 863–864. 10.1093/bioinformatics/btr026 21278185 PMC3051327

[B59] Secades P. Álvarez B. Guijarro J. A. (2001). Purification and characterization of a psychrophilic, calcium-induced, growth-phase dependent metalloprotease from the fish pathogen *Flavobacterium psychrophilum*. Appl. Environ. Microbiol. 67, 2436–2444. 10.1128/AEM.67.6.2436-2444.2001 11375148 PMC92892

[B60] Secades P. Álvarez B. Guijarro J. A. (2003). Purification and properties of a new psychrophilic metalloprotease (Fpp2) in the fish pathogen *Flavobacterium psychrophilum*. FEMS Microbiol. Lett. 226, 273–279. 10.1016/S0378-1097(03)00599-8 14553922

[B61] Solís C. Poblete-Morales M. Cabral S. Valdés J. Reyes A. Avenda-o-Herrera R.. (2015). Neutrophil migration in the activation of the innate immune response to different *Flavobacterium psychrophilum* vaccines in zebrafish (*Danio rerio*). J. Immunol. Res. 2015, 1–9. 10.1155/2015/515187 25815347 PMC4359811

[B62] Starliper C. E. (2011). Bacterial coldwater disease of fishes caused by *Flavobacterium psychrophilum*. J. Adv. Res. 2, 97–108. 10.1016/j.jare.2010.04.001

[B63] Strepparava N. Wahli T. Segner H. Polli B. Petrini O. (2012). Fluorescent *in situ* hybridization: a new tool for the direct identification and detection of *F. psychrophilum*. PLoS ONE 7:e49280. 10.1371/journal.pone.0049280 23152887 PMC3494677

[B64] Sundell K. Wiklund T. (2011). Effect of biofilm formation on antimicrobial tolerance of *Flavobacterium psychrophilum*. J. Fish Dis. 34, 373–383. 10.1111/j.1365-2761.2011.01250.x 21488905

[B65] Suzuki R. Shimodaira H. (2006). Pvclust: an R package for assessing the uncertainty in hierarchical clustering. Bioinformatics 22, 1540–1542. 10.1093/bioinformatics/btl117 16595560

[B66] Takahashi H. Kamakura H. Sato Y. Shiono K. Abiko T. Tsutsumi N.. (2010). A method for obtaining high quality RNA from paraffin sections of plant tissues by laser microdissection. J. Plant. Res. 123, 807–813. 10.1007/s10265-010-0319-4 20221666

[B67] Vatsos I. N. Thompson K. D. Adams A. (2001). Adhesion of the pathogen *Flavobacterium psychrophilum* to unfertilized eggs of rainbow trout (*Oncorhynchus mykiss*) and n-hexadecane. Lett. Appl. Microbiol. 33, 178–182. 10.1046/j.1472-765x.2001.00980.x 11555199

[B68] Venturi V. Ahmer B. M. M. (2015). Editorial: LuxR Solos are becoming major players in cell-cell communication in Bacteria. Front. Cell. Infect. Microbiol. 5:89. 10.3389/fcimb.2015.00089 26649284 PMC4664662

[B69] Verma V. Prasad Y. (2014). Isolation and immunohistochemical identification of *Flavobacterium psychrophilum* from the tissue of catfish, *Clarias batrachus*. J. Environ. Biol. 35, 389–393. 24665767

[B70] Warnes G. R. Bolker B. Bonebakker L. Gentleman R. Liaw W. H. A. Lumley T. (2013). Various R Programming Tools for Plotting Data. Package ‘gplots,’ version 3.0.1. Vienna: Comprehensive R Archive Network, Institute for Statistics and Mathematics Available online at: https://cran.r-project.org/web/packages/gplots/index.html

