# Supplementary material for: Different Phenotypes of Mature Biofilm in Flavobacterium psychrophilum Share a Potential for Virulence That Differs from Planktonic State
Source: Front Cell Infect Microbiol. 2017 Mar 15;7:76. doi: 10.3389/fcimb.2017.00076 (PMC5350093; doi:10.3389/fcimb.2017.00076)
Supplement: Supplementary file 1 [file Table1.PDF]

Supplementary Material

Different Phenotypes of Mature Biofilm in *Flavobacterium psychrophilum* Share a Potential for Virulence That Differs from Planktonic State

Héctor A. Levipan[1, 2]* and Ruben Avendaño-Herrera[1, 2, 3]*

*** Correspondence:**

Héctor A. Levipan: h.levipancolil@uandresbello.edu; Ruben Avendaño-Herrera: ravendano@unab.cl

1.1 Supplementary Table 1

Table S1. Primers used to amplify genes encoding proteins with potential roles in virulence of *F. psychrophilum*. Names (based on the locus tags according to the genome of *F. psychrophilum* strain JIP02/86), description of target genes, amplicon length, primer sequences, and annealing temperatures are depicted.

Primer name	Gene description (gene symbol)	Amplicon (bp)	Sequence (5' to 3')	Annealing temperature (º C)
FP0063-F<br>FP0063-R	Putative hemolysin (FP0063)	215	CTTAATGAAATTTATGACAA<br>TCTTAAAATTAGGTTCTCTT	55
FP0097-F<br>FP0097-R	Flavomodulin (*fmo*)	152	ATATTACAACCTCAGAAAGT<br>TTTGTCTAAACGATATTAAA	52
FP0232-F<br>FP0232-R	Psychrophilic metalloprotease Fpp2 precursor (*fpp2*)	200	ATTTGAAAACTTCTCTATGT<br>ATACTAAATAAATTCCTGCT	52

FP0595-F	Protein of unknown function precursor; putative adhesine (FP0595)	223	CTTTTCAAAACAGTAACATA	55
FP0595-R			GTTAATAACTTTCCAAATCT	
FP1830-F	Protein of unknown function precursor; putative adhesine (FP1830)	192	AAACTCGAGTATATTGTCTT	52
FP1830-R			CCATTAGGATTATAAGGTAT	
FP2019-F	Flavobacterial-specific protein antigen FspA precursor (*fspA*)	161	AAAGTTCTATTTTAGTTGCT	55
FP2019-R			ACTGGTTTCAGTATTATTTC	
