# Supplementary material for: Different Phenotypes of Mature Biofilm in Flavobacterium psychrophilum Share a Potential for Virulence That Differs from Planktonic State
Source: Front Cell Infect Microbiol. 2017 Mar 15;7:76. doi: 10.3389/fcimb.2017.00076 (PMC5350093; doi:10.3389/fcimb.2017.00076)
Supplement: Supplementary file 2 [file Image1.PDF]

Supplementary Material

Different Phenotypes of Mature Biofilm in *Flavobacterium psychrophilum* Share a Potential for Virulence That Differs from Planktonic State

Héctor A. Levipan[1, 2]* and Ruben Avendaño-Herrera[1, 2, 3]*

*** Correspondence:**

Héctor A. Levipan: h.levipancolil@uandresbello.edu

Ruben Avendaño-Herrera: ravendano@unab.cl

1.1 Supplementary Figure 1

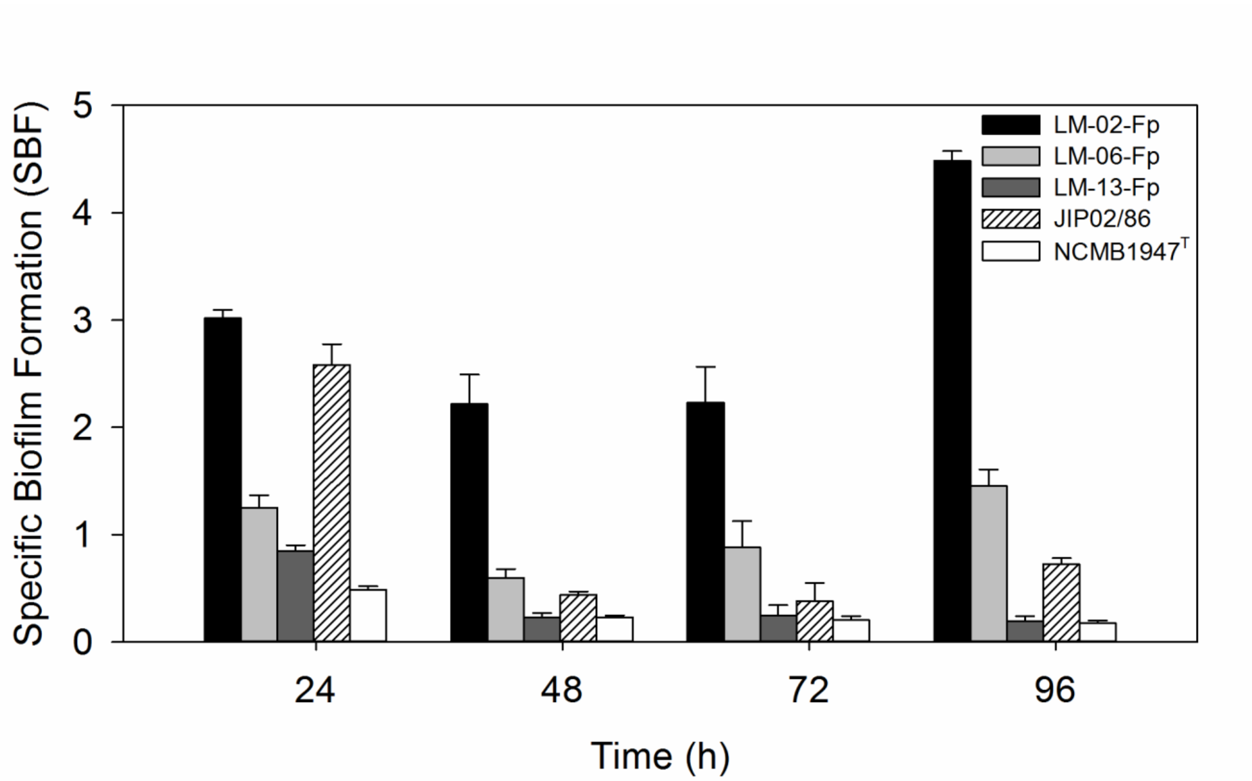

Supplementary Figure 1. Biofilm formation by different *F. psychrophilum* strains. The LM-02-Fp and NCMB1947^{T} strains were the strongest and weakest biofilm producers, respectively. Results are representative of three independent experiments.

1.2 Supplementary Figure 2

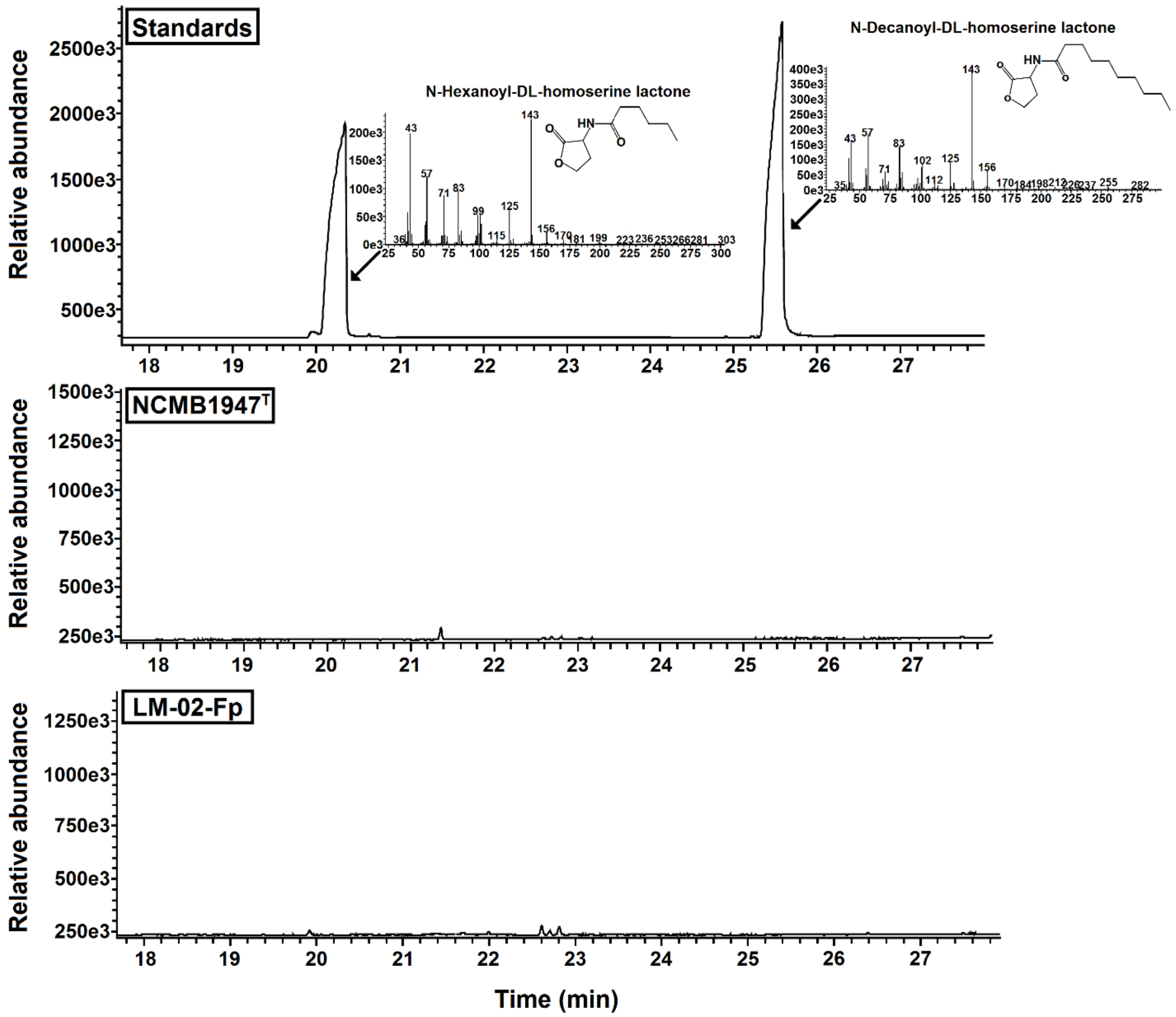

Supplementary Figure 2. Gas chromatograph with mass spectrometry. AHLs were undetected by GC in cell-free culture supernatants of *F. psychrophilum* strains. The strains NCMB1947^T and LM-02-Fp are shown as examples.
